# Quantum Dots-Based Immunochromatographic Strip for Rapid and Sensitive Detection of Acetamiprid in Agricultural Products

**DOI:** 10.3389/fchem.2019.00076

**Published:** 2019-02-28

**Authors:** Ying Liu, Ying Zhao, Tianyi Zhang, Yunyun Chang, Shuangjie Wang, Rubing Zou, Guonian Zhu, Lirong Shen, Yirong Guo

**Affiliations:** ^1^Zhejiang Key Laboratory for Agro-Food Processing, Department of Food Science and Nutrition, Zhejiang University, Hangzhou, China; ^2^Ministry of Agriculture Key Laboratory of Molecular Biology of Crop Pathogens and Insects, Institute of Pesticide and Environmental Toxicology, Zhejiang University, Hangzhou, China

**Keywords:** immunochromatographic strip, quantum dot, acetamiprid, monoclonal antibody, agricultural product

## Abstract

In this study, a rapid and sensitive immunochromatographic strip (ICS) assay, based on quantum dots (QDs), was developed for the qualitative and quantitative detection of acetamiprid in agricultural samples. Acetamiprid-ovalbumin conjugates (ACE-OVA) and goat anti-mouse IgG were sprayed onto a nitrocellulose membrane as a test and control line. Two kinds of anti-acetamiprid monoclonal antibodies (mAb) obtained in our lab were characterized by the ELISA and surface plasmon resonance assay. The competitive immunoassay was established using a QDs-mAb conjugate probe. The visual detection limit of acetamiprid for a qualitative threshold was set as 1 ng/mL to the naked eye. In the quantitative test, the fluorescence intensity was measured by a portable strip reader and a standard curve was obtained with a linear range from 0.098 to 25 ng/mL, and the half maximal inhibitory concentration of 1.12 ng/mL. The developed method showed no evident cross-reactivities with other neonicotinoid insecticides except for thiacloprid (36.68%). The accuracy and precision of the developed QDs-ICS were further evaluated. Results showed that the average recoveries ranged from 78.38 to 126.97% in agricultural samples. Moreover, to test blind tea samples, the QDs-ICS showed comparable reliability and a high correlation with ultra-performance liquid chromatography-tandem mass spectrometry. The whole sample detection could be accomplished within 1 h. In brief, our data clearly manifested that QDs-ICS was quite qualified for the rapid and sensitive screening of acetamiprid residues in an agricultural product analysis and paves the way to point-of-care testing for other analytes.

## Introduction

Acetamiprid ((1*E*)-*N*-[(6-chloro-3-pyridinyl) methyl]-*N*′-cyano-*N*-methyl ethanimidamide), a commercial broad-spectrum chloronicotinyl neonicotinoid insecticide, could inhibit the activity of the nicotinic acetylcholine receptor (nAChR), which leads to system paralysis and the death of insects. Due to its unique mode of action, acetamiprid is more competitive than conventional insecticide. In recent years, it has been considered as an excellent replacement, by the Environmental Protection Agency (EPA), to the organophosphorus insecticides, and it was also considered a good succedaneum of urethane and synthetic pyrethroid pesticides, which had no effect on some sucking and biting insect pests (Ihara et al., 2008; Matsuda et al., 2009; Wang et al., 2013). However, due to the inevitable use in the agricultural industry, acetamiprid might accumulate on the soil surface, which acts as source of contamination for the air and groundwater (Yao and Min, 2006; Gupta and Gajbhiye, 2007). Gradually, the accumulation of acetamiprid in the environment led to the potential toxicity of some mammals and humans through food chains (Pramanik et al., 2006; Sanyal et al., 2008). Maximum residue limits (MRLs) for acetamiprid have been suggested in official legislations from different countries. For instance, the MRLs in apple, cabbage, grain, and tea are 1.0, 3.0, 5.0, and 50.0 mg/kg respectively set by EPA in U.S^1^. Lower values of 0.8, 1.0, 0.5, and 10 mg/kg are seen in China [National food safety standard-maximum residue limits for pesticides in food (GB 2763-2016)]. Moreover, increasingly strict MRLs in the EU, set to 0.8, 1.5, 0.01, and 0.05 mg/kg, is worthy of special attention^2^. Therefore, it is extremely urgent to establish an efficient, sensitive, and economical method with an ultralow detection limit for the *in-situ* measurement of acetamiprid residue in environmental samples and agricultural products.

Traditional methods are competent in determining acetamiprid residues, through instrumental analysis tools such as high-performance liquid chromatography (HPLC) (Obana et al., 2002; Zhou et al., 2006), gas chromatography-mass spectrometry (GC-MS) (Mateu-Sanchez et al., 2003), and liquid chromatography-mass spectrometry (LC-MS) (Yeoh and Chong, 2012). Although being accurate and reliable, these detection methods are time-consuming and costly, relying on expensive instruments and advanced technicians. In the last two decades, immunoassay was proven as a landmark method for pesticide monitoring, due to its advantages in rapid, high-throughput, and on-site screening tests (Liu et al., 2016). In the early Twenty-First century, enzyme-linked immunosorbent assay (ELISA) was established to detect acetamiprid residue by Eiki Watanabe et al. based on the monoclonal antibody (mAb) of acetamiprid with the half maximal inhibitory concentration (IC_50_) of 1.0 ng/mL (Wanatabe et al., 2001) and 0.6 ng/mL (Watanabe et al., 2006). The instrumental assays and ELISA were mainstream until the aptamers targeting acetamiprid were developed. Combined with sensor technology, the aptamers were used in different kinds of sophisticated platforms (He et al., 2011). Generally, electrochemical aptasensors (Fan et al., 2013; Taghdisi et al., 2016), aptamer-based colorimetric sensing (Shi et al., 2013; Qi et al., 2016), and fluorescence resonance energy transfer (FRET) based on aptamers and other nanoparticles (Hu et al., 2016; Lin et al., 2016) have been established. Although these methods show superior detection sensitivity, the demand for on-site screening are growing, taking professional conditions, expensive instruments, and time-consuming sample pretreatments of these assays mentioned above into consideration (Duan et al., 2015).

Immunochromatographic strip (ICS) assay, a combination of chromatography and immunochemical reactions, emerged a long time ago, enabling the separation of the reacted product from the unreacted substances, without additional precipitation or washing (Dzantiev et al., 2014). In previous research, gold-nanoparticles (GNPs)-ICS has been recommended for acetamiprid semi-quantitative detection because of its rapidity, convenience, and suitability for on-site analysis. Although the reaction time is only 10 min, the visual limit of detection (LOD) of acetamiprid was 0.5 mg/kg in tea samples (10 ng/mL in acetamiprid standard solution) (Zhao et al., 2016), 0.005 mg/kg in cucumber samples and 0.03 mg/kg in apple samples (1 ng/mL in acetamiprid standard solution) (Liu et al., 2017). However, the competitive GNPs-ICS is always limited in its relatively low sensitivity and narrow detection range, due to the direct colorimetric measurement. As for the assay sensitivity, the common visual GNPs-ICS considers complete discoloration of the test line, whereas the ICS scanning reader usually allows recording of a small decrease of fluorescence intensity of label binding at the test line. Recently, many ICS assays have been developed to detect environmental contaminants, based on new fluorescent nanoparticles such as Quantum dots (QDs), fluorescent dye-based microspheres (Zhang et al., 2016), lanthanide-based microspheres (Zhang et al., 2017), and up-conversion phosphors (Wang P. et al., 2016).

QDs have unique optical properties such as size-tunable emission, broad adsorption, narrow and symmetric photoluminescence spectra, strong fluorescence intensity, and excellent anti-photobleaching property (Huang et al., 2016). QDs are therefore capable of a robust reporter and can develop a highly-sensitive ICS for rapid diagnosis. For instance, tumor markers (Wang et al., 2015), total IgE in human serum (Berlina et al., 2013), were successfully detected by QDs-ICS. In addition, the application of QDs-ICS in agricultural production and food safety monitoring has been rising. The detection of general mycotoxins like aflatoxin B (Ren et al., 2014) and zearalenone (Duan et al., 2015), several antibiotics in milk products (Taranova et al., 2015), and some biomarkers of organophosphorus agents (Zou et al., 2010; Wang et al., 2011; Zhang et al., 2013) have been achieved. Nevertheless, there are few reports on the QDs-ICS test for neonicotinoid pesticides (Wang et al., 2017), and no studies on the QDs-ICS assay, used for acetamiprid detection have been published.

In this study, a specific and high-affinity mAb toward acetamiprid was screened and characterized by an indirect competitive ELISA (ic-ELISA) and a surface plasmon resonance (SPR) assay. Based on QDs and the developed mAb, a novel ICS was established for the qualitative and quantitative detection of acetamiprid. A portable strip reader was used to record the fluorescent intensity of the test (FI_T_) and control (FI_C_) lines. Various parameters were optimized, and the performance of ICS was evaluated. Furthermore, the practicability of QDs-ICS was valued by detecting acetamiprid residues in real samples, compared with GNPs-ICS and UPLC-MS/MS. Our results indicate that the developed QDs-ICS is a rapid, portable, sensitive, and economical assay for laboratory tests or on-site screenings of acetamiprid.

## Materials and Methods

### Materials and Reagents

ZnCdSe/ZnS QDs nanocrystals modified with PEG and carboxyl groups (Q3605) were obtained from JiaYuan Quantum Dots Co., Ltd. (Wuhan, China). Acetamiprid and other neonicotinoids pesticide standards were obtained from the Agro-Environmental Protection Institute, Ministry of Agriculture (Tianjin, China). Goat anti-mouse IgG was purchased from Jisen Biotech Company (Beijing, China). The amine coupling kit for SPR assay contains 0.1 M N-hydroxysuccinimide (NHS), 0.4 M N-(3-dimethylaminopropyl)-N′-ethylcarbodiimide hydrochloride (EDC) and 1 M ethanolamine-HCl (pH 8.5) from GE Healthcare (USA). EDC powder for QDs/mAb conjugation was supplied by Sigma-Alorich (St Louis, MO, USA). Sucrose and methanol were supplied by Sinopharm Chemical Reagent (Shanghai, China). Tween 20, D- (+)—trehalose anhydrous, and polyvinyl pyrrolidone (PVP) were purchased from Aladdin (Shanghai, China). Ovalbumin (OVA) and bovine serum albumin (BSA) were obtained from VWR (Amresco, USA). All other chemicals were standard products of analytical grade.

Some other materials were as follows: CM7 sensor chip (GE Healthcare, Madison, USA); 50 kDa ultra-filtration tube (Millipore, USA); Size exclusion chromatography filling (Superdex G200, GE Healthcare, USA); Gel chromatography column for purification (Thermo, USA). Different specifications of nitrocellulose (NC) membrane (Millipore, USA & Sartorius, Germany & Pall, USA); Glass fiber membranes (Sartorius, Germany); Filter paper and semi-rigid polyvinyl chloride (PVC) sheets (Jiening Biological Technology Co., Ltd., Beijing, China). Female Balb/c mice and F1 hybrid mice were provided by the Shanghai Lab Animal Research Center (China).

Buffers in SPR tests such as phosphate buffer saline (PBS, 0.01 M, pH 7.4) with 0.05% (v/v) polysorbate surfactant P20 (PBS-P+), 10 mM sodium acetate (pH 4.5, 5.0, and 5.5), and regeneration buffers were obtained from GE Healthcare Bioscience AB (Upsala, Sweden). Carbonate buffer saline (CBS, 50 mM, pH 9.6), PBS (10 mM, pH 7.4), and PBST (10 mM PBS containing 0.05% tween-20, pH 7.4) were prepared for ELISA. Ten millimolar Borate buffer (BB, pH 7.4), PBS (pH 7.4), Tris-HCl (pH 7.4), and deionized water served as working buffers for ICS tests.

### Instruments

ChemiDocTMMP imaging system with Image lab 5.2 analysis software (Bio-RAD, USA); SPR technique on Biacore T200 biosensor system (GE Healthcare, Madison, USA) with data acquisition software (Biacore T200 Evaluation Software Version 3.0); AcquityUltra Performance LC (Waters, Milford, MA, USA) with Applied Biosystems Triple Quad 5500 (ESI–MS/MS; Foster, CA, USA) in electrospray negative-ion multiple reaction modes; Ultrasonic cleaner (Ningbo Scientz Biotechnology Co., LTD., China); Milli-Q purification system (Millipore, Bedford, USA); Index Cutter-I slitter (Grand Island); IsoFlow dispenser (Imagene Technology, USA); The laser flashlight and the portable fluorescence strip reader (365 nm) were all purchased from Jiening Biological Technology (Beijing, China).

### Preparation and Characterization of QDs-mAb Conjugate

The anti-acetamiprid mAb was screened and characterized by ic-ELISA and SPR assay. A detailed presentation is shown in the Supplementary Material. The QDs-mAb conjugate was prepared using the activated ester method. The carboxyl groups on the QDs' surface were activated by EDC, and then reacted with amine groups of anti-acetamiprid mAb. Briefly, 200 μL of Q3605 (8 μM) was diluted in 738 μL of reaction buffer (BB, 10 mM, pH 7.2), in which anti-acetamiprid mAb (3.89 mg/mL) was subsequently added. The mixture was gently stirred at room temperature (RT, 25°C) for 5 min. Thereafter, 62 μL of EDC (10 mg/mL, in 10 mM BB) was added into the mixture of Q3605 for a final concentration of 1 μM. After a further 3 h stirring in RT, the resulting mixture was centrifuged at 12,000 rpm for 3 min and concentrated to 200 μL using a 50 kDa ultrafiltration tube. A gel chromatography column for purification was packed with Superdex G200. Three quarters of the volume passing column were collected and stored in 400 μL of preservation solution. In order to obtain a relative economical dosage of materials and more stable QDs-mAb probes, the coupling molar ratio and preservation solution should be optimized and checked by comparing the fluorescence intensity of QDs-ICS. After optimization, 10 mM BB with 1% (w/v) BSA, 0.05% (w/v) PVP, 0.05% NaN_3_ and 0.2% (w/v) trehalose was used for the stock solution at 4°C.

### Assembly of QDs-ICS

As shown in Figure S3, QDs-ICS was made out of four parts: a sample pad (glass fiber membranes), a NC membrane, an absorbent pad (filter paper), and a PVC sheet. Sample pads should be pretreated with PBST containing 0.25% (w/v) BSA, 0.25% (w/v) PVP, and 1% (w/v) trehalose. The ACE-OVA conjugate (1 mg/mL) and goat anti-mouse IgG (0.2 mg/mL) were sprayed, respectively, onto NC membranes as the test (T) and control (C) lines, and the distance between them was about 5 mm. All components of ICS were dried at 37°C overnight. The sample pad, NC membrane, and absorbent pad were attached to the PVC sheet, followed by cutting into 3 mm-wide test strips and packing into plastic casings for storage in a dry space at 4°C.

### ICS Test Procedure

**Figure 1:**
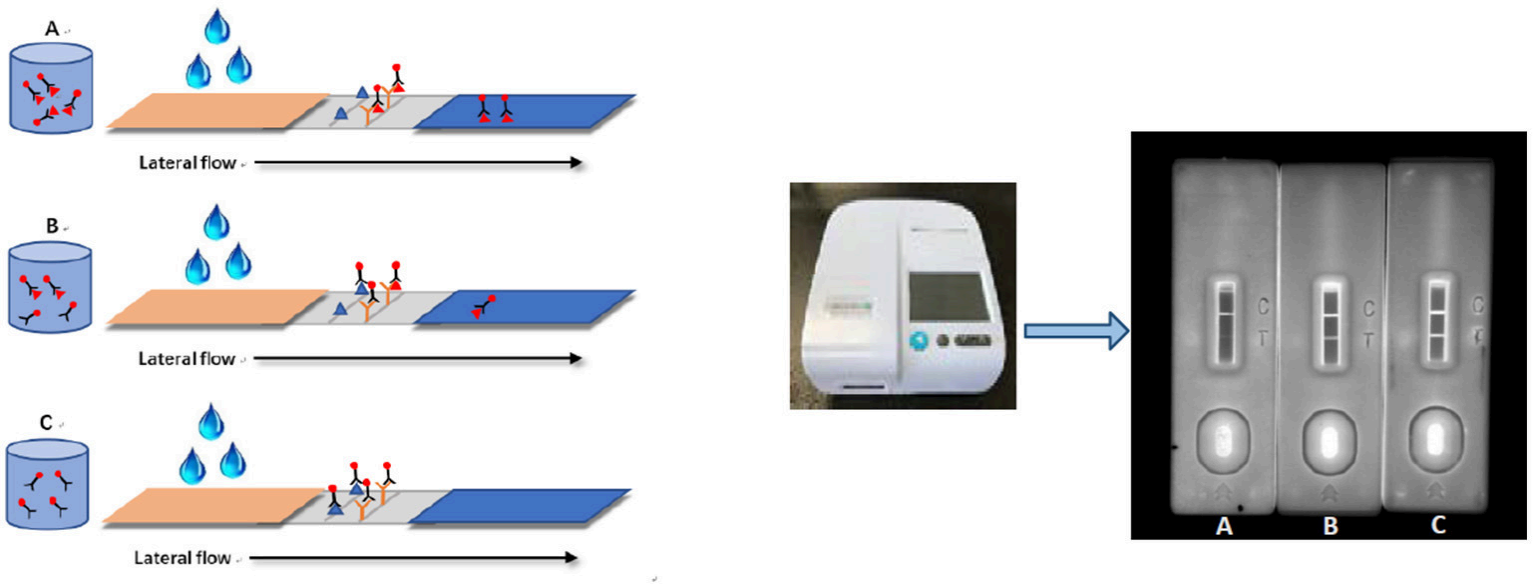
Schematic illustration of QDs-ICS for qualitative detection of acetamiprid. Panels **(A,B)** are the results of positive samples which are spiked with 10 and 1 ng/mL acetamiprid. Panel **(C)** is the result of negative sample.

As a competitive reaction, the QDs-Ab probe (25 μL) and analyte solution (25 μL) were premixed at room temperature for 5 min, and then added onto the sample pad. The QDs-Ab could react with acetamiprid (if it existed in the analyte solution). With the pull of the absorbent pad, the mixture passed through the NC membrane for 20 min. During the crucial process, the unreacted QDs-Ab was captured by the T line, and subsequently the immunocomplex reacted with the C line, which resulted in a strong fluorescence on the T and C lines under 365 nm irradiation, respectively. Under the circumstance of a stable fluorescence intensity in the C line, the more acetamiprid in the sample solution, the lower the fluorescence intensity appears on the T line (Figure 1). Judgement with the naked eye, with goggles under 365 nm of excitation light, only the C line changed to red for the positive sample, but the negative one resulted in two red lines except for the invalid ones without red C lines.

The qualitative performance of ICS was evaluated with the threshold level for the detection of acetamiprid. According to ICS procedure, the lowest concentration of acetamiprid that caused complete invisibility on the T line was confirmed as the threshold level. For quantitative detection, the fluorescence intensity of the T (FI_T_) and C line (FI_C_) should be tested by the portable fluorescence strip reader. The standard curve was established by plotting the FI_T_/FI_C_ × 100% against the logarithm of the gradient target analyte concentration. The concentration of acetamiprid in the detected samples could be calculated by a linear equation based on the inhibition rates and the corresponding standard concentrations.

### Specificity of the ICS

Seven other neonicotinoid pesticides were tested by the optimized reaction system to verify the specificity of the developed ICS. The standard analyte solutions were prepared by spiked stock solutions in BB, at the optimized circumstances, to a final concentration of 0.1, 0.5, 1.0, 2.0, 5.0, 10.0, 20.0, 50.0, 100.0, 200.0 ng/mL. As above, for semi-quantitative detection, the lowest concentration of the analyte that caused complete invisibility on the T line was confirmed as the visual sensitivity.

### Method Validation

Six kinds of samples were tested by the developed ICS. Brown rice samples were collected from paddy fields (Hangzhou, China). Cabbage and apples were purchased from Walmart supermarket (Hangzhou, China). Black, green and oolong tea were obtained by Tea Research Institute, Chinese Academy of Agricultural Sciences (Hangzhou, China). These samples were considered to be acetamiprid-free by UPLC-MS/MS.

According to different eating habits, the pretreatments were carried out as follows. The homogenized cabbage (5 g), apples (5 g), and powdered rice (5 g) samples were spiked with three final concentrations of the acetamiprid standard (0.004, 0.02, and 0.1 mg/kg) dissolved in methanol. In particular, the spiked rice needed to be soaked in 10 mL water for 10 min. After incubation for 2 h at RT, 10 mL acetonitrile was added to each agricultural sample and vigorously shaken for 10 min. Then, NaCl (1 g) and MgSO_4_ (5 g) were added to the mixture. After 10 min centrifugation at 4,000 × g, 2 mL supernatant was respectively dried and re-dissolved in BB for matrix effect detection or recovery tests. As for the tea samples, six gradient acetamiprid standards (final concentration 0.02, 0.04, 0.1, 0.2, 0.5, and 1 mg/kg) were added in the black tea (1 g), green tea (1 g), and oolong tea (1 g), respectively. After 2 h incubation, the spiked teas were brewed in 50 mL boiling water, and then tested by QDs-ICS. In this study, gradient dilutions (1:2, 1:5, 1:10, 1:20) of extraction from rice, apple, and cabbage samples and gradient dilutions (1:50, 1:100, 1:200, 1:400) of extraction from green, black, and oolong tea samples were prepared by BB. Acetamiprid standard solutions were individually prepared in a matrix extract with BB as the control.

### Detection of Authentic Tea Samples by QDs-ICS, GNPs-ICS, and UPLC-MS/MS

Ten types of tea products purchased from local supermarket were screened by the current QDs-ICS and the developed GNPs-ICS (Zhao et al., 2016) to detect acetamiprid residue. The pretreatment of samples were performed as mentioned above.

In order to ensure the accuracy of the tests, results should be identified by UPLC-MS/MS. The instrument used was composed of an UPLC system, coupled with a tandem mass spectrometer (equipped with ESI interface in positive mode at 5,500 V, 600 C). The analytical column was ACQUITY UPLC® HSS T3 column (2.1 × 100 mm, with a 1.8μm particle size; Waters). The isocratic eluent included acetomitrile and 0.1% formic acid (95:5, v/v) at the flow rate of 300 μL/min with 10 μL sample injection volume. The analysis was performed on multiple reactions monitoring (MRM) mode. Two qualifying ions (223 → 126 amu and 223 → 90 amu) were selected and the parameters of mass spectrometer were set as Table S7.

## Results and Discussion

### Preparation and Characterization of Anti-acetamiprid mAb

Two murine hybridoma cell lines, ACE-A6 and ACE-G7, secreting anti-acetamiprid mAbs were obtained by stepwise selection. The mAbs' performances of recognizing the target pesticides were characterized by ic-ELISA and SPR. Through the results of ic-ELISA, the mAb from ACE-A6 obtained higher affinity binding acetamiprid, but the mAb from the ACE-G7 was more sensitive to thiacloprid (Figure S1). However, the differences in binding affinity were relatively invisible (Figure S2 and Table S1). mAb against acetamiprid commonly shows more or less cross-reactivity with thiacloprid, according to reported conclusions (Watanabe et al., 2013; Liu et al., 2017). This phenomenon could be explained by the common chloropyridine ring in their structures. Here, ACE-A6 was chosen to use in the following tests.

### Preparation and Characterization of QDs-Ab Conjugate

The QDs-Ab probe was obtained by coupling ACE-A6 with Q3605 using an active ester method. The selection of ACE-A6 is explained in the Supplementary Material. As shown in Table 1, when the content of ACE-A6 was at the same level, the values of FI_T_ and FI_C_ were the highest with the molar ratio of 1:10 (Q3605: mAb). At the same time, the ratio of the FI_T_/ FI_C_ value was close to 1.0, which showed the same color intensity in negative conditions as observed by the naked eye. Importantly, the formulation of the stock solution should be optimized for stable storage of QDs-Ab. The QDs-Ab probes which were kept in different stock solutions, with various inclusions, were used in ICS and the results are presented in Table S2. Obviously, the stock solution with 1% BSA, 0.2% trehalose, 0.05% PVP, and 0.05% NaN_3_ showed greatest stability and homogeneity with the strongest fluorescence intensity.

**Table 1:** Optimization of the molar ratio of Q3605-mAb conjugation.

**The molar ratio of conjugation (Q3605: mAb)**	**Dilution times of QDs-Ab**	**FI_**T**_ value**	**FI_**C**_ value**	**FI_**T**_/FI_**C**_ value**
1: 0.5	50	5	655	0.008
1: 1	100	1,289	2,805	0.460
1: 2	200	1,794	3,299	0.544
1: 5	500	1,818	2,675	0.679
1: 10	1000	2,131	2,286	0.932
1: 20	2000	1,077	1,022	1.054

**Figure 2:**
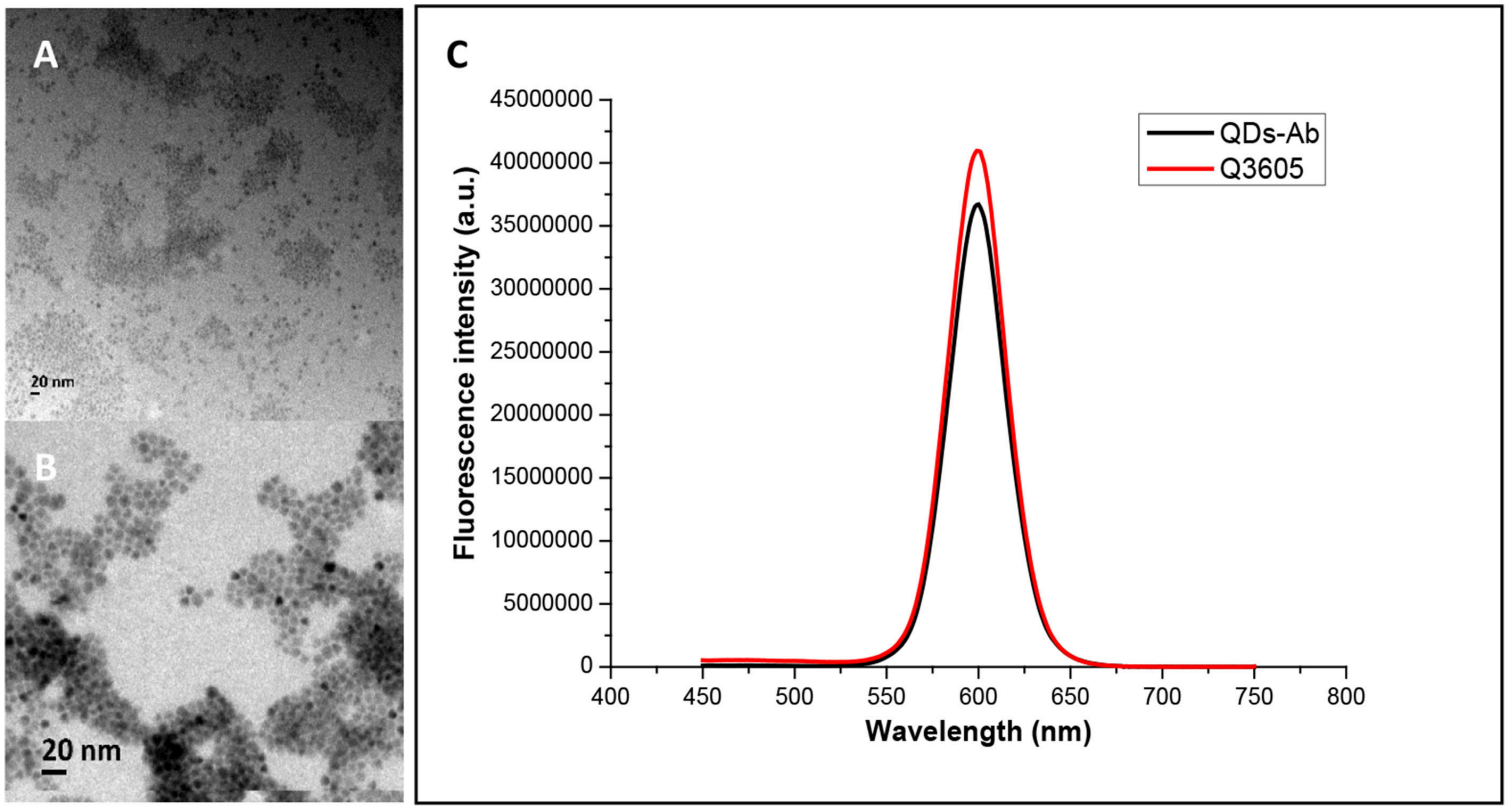
Characterization of QDs. **(A)** High-resolution TEM image of Q3605 (size 8–9 nm). **(B)** High-resolution TEM image of QDs-Ab conjugate (size 11–12 nm). **(C)** The fluorescence intensities of Q3605 and QDs-Ab (emission maximum 600 nm).

As shown in Figure 2C, a QDs-Ab probe was synthesized in the optimized conditions, and the maximum emission wavelengths of Q3605 and QDs-Ab conjugate were monitored. Compared to bare QDs, QDs-Ab conjugate showed a slightly higher fluorescence intensity in a same maximum emission wavelength. Figures 2A,B showed the clear and uniform particles in TEM images.

### Establishment of QDs-ICS

Under the optimal conditions, the Q3605s were successfully conjugated with anti-acetamiprid mAb ACE-A6. In order to improve the stability and sensitivity of ICS, some parameters should be optimized orderly as shown in the Supporting Information (“Assembly and Optimization of QDs-ICS”). The optimized condition for acetamiprid analysis by ICS were as follows: the selected NC membrane (Sartorius CN140) and the sample pad (blocked by 0.25% (w/v) BSA, 0.25% (w/v) PVP, and 1% (w/v) trehalose) were fitted and dried together; the T line (ACE-OVA) and C line (goat anti-mouse IgG) were diluted by PBST (containing 10% sucrose) and sprayed on the NC membrane; the QDs-Ab and the standard (or sample) were diluted by BB (10 mM, pH 7.2) and pre-reacted for 5 min; 50 μL pre-incubated mixture was added onto the sample pad for testing in RT. After 20 min, the results were recorded by the portable reader.

### Assay Sensitivity and Specificity

**Figure 3:**
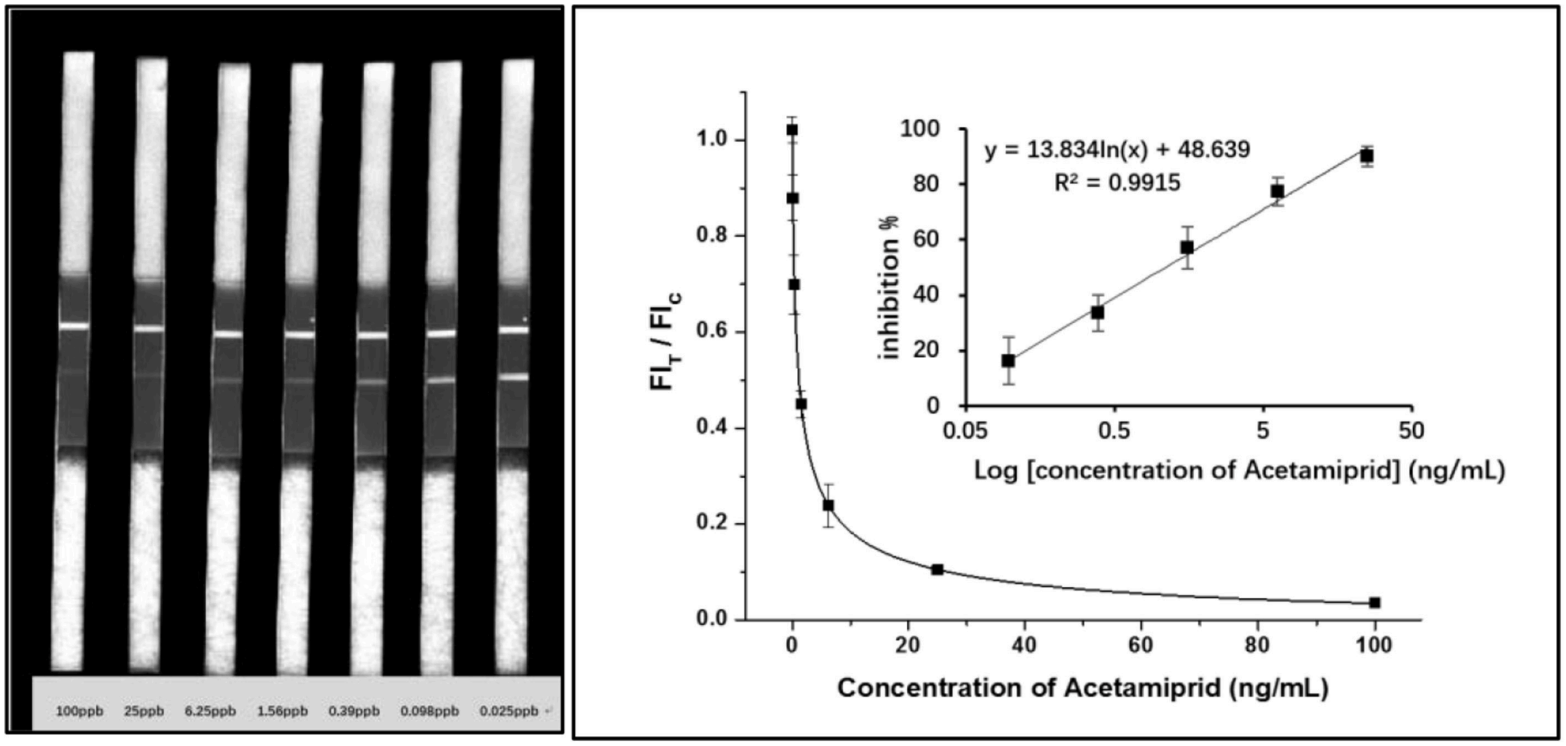
Calibration curves obtained from gradient acetamiprid-spiked standards and relevant results of QDs-ICS.

To determine the sensitivity of the assay, a standard curve for optimal ICS was constructed by plotting the ratio of FI_T_/FI_C_ against acetamiprid concentrations. The standard solutions were prepared by diluting acetamiprid stock solution (0.1 mg/mL) with BB to final concentrations of 0.024, 0.098, 0.39, 1.5625, 6.25, 25, and 100 ng/mL, respectively. We calculated the inhibition ratio by comparing the fluorescence corresponding to each concentration of acetamiprid to the negative BB. The linear relation of the inhibition ratio and the logarithm of acetamiprid were presented aside (*n* = 5). Seen from the calibration curves in Figure 3, the linear regression equation was *y* = 13.834 ln (X) + 48.439 (*R*^2^ = 0.9915), with IC_50_ of 1.12 ng/mL similar to that of icELISA in this study (1.76 ng/mL, Figure S1). The linear range of GNPs-ICS ranged from 0.098 to 25 ng/mL. Such a wide detection range with 3 orders of a magnitude helps to rapidly screen blind samples with an unknown level of acetamiprid concentration. The LOD was therefore set as the lowest concentration of the linear range, at 0.098 ng/mL (about 0.44 nM), which was close to or even lower than the values detected by some aptasensors and FRET assays in the literature (Table 2).

**Table 2:** Comparison of the proposed ICS and other methods for acetamiprid detection in analytical performances.

**Method**	**Visual or Instrumental detection**	**Bio-recognition element**	**LOD (nM)**	**Linear range (nM)**	**Tested samples**	**References**
Aptamer-based EIS	Instrumental detection	Aptamer	1	5–600	Wastewater/tomato	Fan et al., 2013
Aptamer-based resonance light scattering	Instrumental detection	Aptamer	1.2	0–100	Lake water	Wang C. et al., 2016
Aptamer-based colorimetric method	Instrumental detection	Aptamer	5	75–7,500	Soil	Shi et al., 2013
FRET (QDs/MWCNTs)	Instrumental detection	Aptamer	0.7	0–150	River water/cabbage	Lin et al., 2016
FRET (UCNPs/GNPs)	Instrumental detection	Aptamer	3.2	50–1,000	Tea	Hu et al., 2016
FRET (QDs/GNPs)	Instrumental detection	Aptamer	7.29	50–1,000	Vegetable	Guo et al., 2016
Direct competitive ELISA	Instrumental detection	mAb	1.4	1.4–56.1	Fruit/vegetable	Wanatabe et al., 2001
Direct competitive ELISA	Instrumental detection	mAb	0.24	0.8–13.5	Fruit/vegetable	Watanabe et al., 2006
GNPs-ICS	Visual detection	mAb	44.9	/	Tea	Zhao et al., 2016
GNPs-ICS	Visual detection	mAb	4.5	/	Cucumber/apple	Liu et al., 2017
QDs-ICS	Instrumental detection	mAb	0.44	0.44–112.3	Tea/rice/apple/cabbage	This study

**Figure 4:**
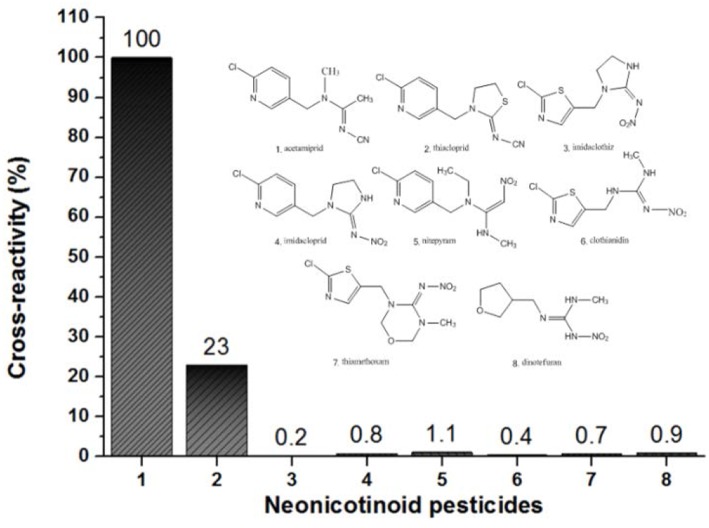
Cross-reactivity of QDs-ICS for acetamiprid toward seven other neonicotinoid pesticides.

Additionally, the visual LOD was performed as the lowest concentration of acetamiprid which caused invisibility on the T line, as observed by the naked eye, namely, the visual sensitivity was around 1 ng/mL for qualitative detection. In our previous study, the ACE-A6 mAb was used to establish GNPs-ICS and the visual LOD was 10 ng/mL (Zhao et al., 2016). It is obvious that QDs-ICS shows higher sensitivity than GNPs-ICS when using the same mAb. Moreover, the specificity of ICS was tested by evaluating its reactivity with other seven neonicotinoid pesticides (Figure 4). It was found that the ICS had no cross-reactivity with these compounds expect for thiacloprid (36.68%). This result was in agreement with the findings of the ic-ELISA and SPR assays mentioned above.

### Assay Validation

#### Matrix Effect

As a kind of rapid portable method for residue detection, ICS has common challenges in the pretreatment of samples. The complex matrix, containing not only pigment but also a high number of saccharides, polyphenols and proteins can interfere with the determination of analytes. These interferences can be reduced in various ways, among which dilution with BB (or water on site) is a simple and effective procedure, although it may simultaneously cause desensitization. Usually, the matrix effect is evaluated by comparing the standard curves obtained from the working buffer and sample extraction solution. It can be determined using the equation, matrix effect (%) = (1–slope_matrix_/slope_buffer_) × 100%, and if it is in the range between −20 and 20%, the matrix effect can be ignored.

According to the ICS protocol, the detection of the acetamiprid standard, which is prepared by several dilution times of the extraction from six kinds of products, proceeded. The calibration curves were established, and then the matrix effects were calculated. Seen from Table S6, 10-fold dilution time could reduce the matrix effect from rice, apples, and cabbage samples, while 100-fold was required to minimize the influence from green, black, and oolong tea because of their complicated matrices.

#### Recovery Test

To estimate the reliability of ICS, the recovery test was performed by adding several concentrations of acetamiprid standard to certain samples, which were quantitatively detected according to the ICS program above. It was noteworthy that the concentrations of acetamiprid standard of the diluted matrix-matched extracts fell into the detection range of ICS to ensure the veracity of the assay. According to the principle, 4, 20, 100 ng/mL of acetamiprid standards were spiked into three agricultural samples and 20, 40, 100, 200, 500, 1,000 ng/mL of acetamiprid were spiked into four tea samples, respectively. As shown in Table 3, the mean recovery ranged from 81.77 to 109.70% in agricultural samples and from 78.38 to 126.97% in tea samples (*n* = 9). Furthermore, the coefficient of variation (CV) varied from 6.09 to 34.00% for the intra-day test and from 7.64 to 31.57% for the inter-day test. Such high variations were probably ascribed to the fact that some QDs-Ab conjugates were appropriate for accumulation, after long-term storage. This problem needs to be solved in the future work.

**Table 3:** Recovery of acetamiprid in spiked samples by QDs-ICS.

**Sample**	**Spiked (mg/kg)**	**Dilution times of matrix extraction**	**Detected **^a^** (ng/mL)**	**Coefficient of variation (%)**		**Mean recovery (%)**
				**Intra-day (*n* = 3)**	**Inter-day (*n* = 3)**	
Rice	0.004	10	0.34 ± 0.05	16.41	15.96	85.11
	0.02		1.84 ± 0.36	19.12	19.55	92.19
	0.1		10.54 ± 1.78	14.22	17.31	105.39
Apple	0.004	10	0.43 ± 0.07	15.01	16.60	107.79
	0.02		2.19 ± 0.29	14.06	13.61	109.70
	0.1		10.72 ± 1.21	12.47	11.95	107.19
Cabbage	0.004	10	0.37 ± 0.12	34.00	31.57	93.36
	0.02		1.64 ± 0.19	11.34	12.54	81.77
	0.1		9.79 ± 1.51	16.20	15.60	97.87
Green tea	0.02	100	0.19 ± 0.02	8.39	12.06	97.03
	0.04		0.51 ± 0.07	14.07	14.04	126.97
	0.1		0.98 ± 0.19	23.34	20.36	97.81
	0.2		1.94 ± 0.40	19.45	20.70	96.98
	0.5		5.91 ± 0.98	13.27	17.36	118.13
	1		11.14 ± 2.46	23.68	22.24	111.41
Black tea	0.02	100	0.16 ± 0.03	11.11	16.22	78.38
	0.04		0.42 ± 0.04	10.47	10.25	104.95
	0.1		1.08 ± 0.25	22.13	23.37	107.86
	0.2		1.96 ± 0.29	12.36	15.48	98.22
	0.5		5.90 ± 0.87	14.23	15.31	118.08
	1		10.78 ± 1.00	8.22	9.00	107.83
Oolong tea	0.02	100	0.24 ± 0.02	8.14	9.31	120.52
	0.04		0.40 ± 0.05	10.18	12.58	101.12
	0.1		0.88 ± 0.27	24.04	30.34	87.70
	0.2		2.36 ± 0.17	8.07	7.64	118.17
	0.5		5.32 ± 0.48	6.09	9.88	106.32
	1		11.02 ± 1.23	10.06	11.37	110.17

a *The values of detected concentrations were from the diluted extraction liquid, presented as mean±standard deviation (n = 9)*.

#### Actual Sample Testing

To verify the accuracy of the assay, 10 tea samples underwent qualitative and quantitative detection by QDs-ICS on the basis of the protocol discussed above. By comparison, GNPs-ICS was used to detect the blind samples. Considering the limitation of ICS, UPLC-MS/MS served as touchstone to evaluate the reliability of the assays. The limit of quantitation of UPLC-MS/MS was 0.1 ng/mL, defined as 10 times the average baseline noise, meeting the examination requirement. The mass spectrogram of 1 ng/mL-spiked acetamiprid in a tea matrix is presented in Figure S5. As listed in Table 4, the quantitative detection results of QDs-ICS were practically satisfactory compared to those of UPLC-MS/MS, and QDs-ICS was more sensitive than GNPs-ICS for visual detection.

**Table 4:** The screening of blind tea samples by GNPs-ICS, QDs-ICS, and UPLS-MS/MS.

**Samples**	**Visual detection**		**Quantitative detection**	
	**GNPs-ICS**	**QDs-ICS**	**QDs-ICS (ng/g)**	**UPLC-MS/MS (ng/g)**
S1	–^a^	+	132	165
S2	–	–	31	20
S3	–	+	159	142
S4	–	–	ND^d^	ND
S5	–	–	6	9
S6	–	–	ND	ND
S7	–	–	63	42
S8	–	–	ND	ND
S9	++^b^	++	Out of range^e^	3250
S10	+^c^	+	712	625

a negative result.

b strongly positive result.

c weakly positive result.

d not detectable.

e exceeds the linear range of detection.

## Conclusions

In this work, the obtained antibodies against acetamiprid were characterized and determined using the ELISA and SPR assay, and subsequently a QDs-Ab conjugate was prepared and an ICS assay was successfully developed for rapid and highly sensitive detection of acetamiprid. It takes < 30 min for sample preparation and 25 min for ICS test. For the acetamiprid standard solution, the optimal QDs-ICS presents a qualitative threshold of 1 ng/mL, as observed by the naked eye, and a quantitative detection limit of 0.098 ng/mL, as observed by the scanning reader, which are more sensitive than previously reported methods. According to the spiked recovery test, the minimum detectable quantity of acetamiprid in tea was 0.02 mg/kg by QDs-ICS, much lower than 0.5 mg/kg by GNPs-ICS (Zhao et al., 2016). In other agricultural samples such as rice, apples, cabbage, the minimum detectable quantities were all 0.004 mg/kg, indicating that developed QDs-ICS can reach the requirement of the strict MRLs set by the EU. In addition, the assay shows satisfying performance with a high correlation to UPLC-MS/MS when testing the blind samples of tea products, in which the acetamiprid residue was tightly controlled. To the best of our knowledge, this is the first report of the qualitative and quantitative detection of acetamiprid using a QDs based lateral flow immunoassay. On account of its significant advantages in rapid, sensitive, economical, on-site screening, and size-tunable fluorescent effect, the proposed ICS method provides an alternative tool for food safety monitoring.

## Ethics Statement

This study on mice was carried out in accordance with the recommendations of the animal welfare committee of Zhejiang University in China.

## Author Contributions

YL carried out experiments, analyzed data, and wrote the manuscript draft. YZ carried out experiments and analyzed data. TZ, YC, RZ, and SW assisted with experiments. GZ and LS gave experimental guidance. YG designed the experiments, analyzed data and revised the manuscript.

### Conflict of Interest Statement

The authors declare that the research was conducted in the absence of any commercial or financial relationships that could be construed as a potential conflict of interest.

## Footnotes

^1^https://www.ecfr.gov/cgi-bin/retrieveECFR?gp=1&SID=329cdd35a9f3fc0210e9e9bdfa4001ec&ty=HTML&h=L&mc=true&r=SECTION&n=se40.26.180_1578

^2^http://ec.europa.eu/food/plant/pesticides/eu-pesticides-database/public/?event=pesticide.residue.CurrentMRL&language=EN
