# Supplementary material for: Quantum Dots-Based Immunochromatographic Strip for Rapid and Sensitive Detection of Acetamiprid in Agricultural Products
Source: Front Chem. 2019 Feb 28;7:76. doi: 10.3389/fchem.2019.00076 (PMC6403152; doi:10.3389/fchem.2019.00076)
Supplement: Supplementary file 1 [file Data_Sheet_1.docx]

Supplementary Material

Quantum dots-based immunochromatographic strips for rapid and sensitive detection of acetamiprid in agricultural products

**Ying Liu^1,2^, Ying Zhao^2^, ^a^ Tianyi Zhang^2^, Yunyun Chang^2^, Shuangjie Wang^2^, Rubing Zou^2^, Guonian Zhu^2^, Lirong Shen^1^*, Yirong Guo^2^***

*^1^ Department of Food Science and Nutrition, Zhejiang Key Laboratory for Agro-Food Processing, Zhejiang University, Hangzhou 310058, China*

*^2^ Institute of Pesticide and Environmental Toxicology, Ministry of Agriculture Key Laboratory of Molecular Biology of Crop Pathogens and Insects, Zhejiang University, Hangzhou 310058, Chi*

†The authors wish it to be known that, in their opinion, the first two authors should be regarded as Joint-First Authors.

* The corresponding authors, E-mail: yirongguo@zju.edu.cn; [shenlirong@zju.edu.cn](mailto:shenlirong@zju.edu.cn).

**Screening of anti-acetamiprid mAb.** Two murine hubridoma cell lines, ACE-A6 and ACE-G7, secreting anti-acetamiprid mAbs were produced by fusing splenocytes from Balb/c mice immunized against an ACE-BSA conjugate with the myeloma cell line (Sp2/0) that was previously developed in our lab. The two cell lines were injected into enterocoelias of F1 hybrid mice to produce ascetic fluids, which were purified as high concentrations of mAbs.

**Characterization of anti-acetamiprid mAb.** The mAbs’ performance of recognizing the target pesticides were characterized by ic-ELISA and SPR and then the mAb, which exhibited higher affinity against acetamiprid, was chosen. Ic-ELISA procedures were described in our previous work (Liu et al.2016). Briefly, the ACE-OVA conjugate was used as the coating detective antigen. The optimum working concentrations of antigen and antibody were determined through chessboard titration. Standard competition curves were acquired by plotting the inhibition rate against the logarithm of the analyte concentration.

Different from the ic-ELISA, the SPR assay was based on a direct affinity between the antibodies and analytes. Firstly, the chip surfaces of the flow cell channel 2 and 4 were covalently activated by injecting a 400 μL mixture of 0.1 M NHS and 0.4 M EDC (1:1, v/v), while the flow cell channel 1 and 3 served as references. Afterward, the two mAb A6 (100 μg/mL) and G7 (50 μg/mL) in the 10 mM sodium acetate buffer (pH 4.5/5.0/5.5) were combined in the chip surface, respectively, while the remaining unreacted esters were blocked with 1 M ethanolamine (pH 8.5) ensuring the that the resonance units (RU) were in the 34000-38000 range. A gradient concentration of acetamiprid were diluted by PBS-P+. The kinetics/affinity measurement was automatically conducted by setting a series of progress of association, dissociation, and regeneration (10 mM glycine-HCl, pH 2.0/2.5/3.0). The pH of the sodium acetate buffer and glycine-HCl should be determined according to the RU value and regeneration capacity. Finally, the data that eliminated the background were analyzed using the Biacore T200 Evaluation Software Version 3.0.

Ic-ELISA was used to determine the sensitivity and specificity of the mAbs of ACE-A6 and ACE-G7. The best working concentration of antigen (ACE-OVA) was 0.5 mg/L, and antibodies were 0.24 mg/L for ACE-A6 and 0.06 mg/L for ACE-G7. As shown in **Figure S1**, both A6 and G7 were able to recognize two kinds of neonicotinoid insecticides, with IC_50_ of 1.76/11.24 ng/mL for acetamiprid and 11.27/1.75 ng/mL for thiacloprid, respectively. Using the results of ic-ELISA, the mAb from ACE-A6 obtained a higher affinity binding acetamiprid, but the mAb from the ACE-G7 was more sensitive to thiacloprid.

Additionally, ic-ELISA results were verified by a SPR assay as shown in **Figure S2**. The SPR data was reliable in that the colored lines matched well with the black curves. **Table S1** respectively lists the binding kinetic values of A6 and G7 against acetamiprid and thiacloprid. The equilibrium dissociation constant (*K_D_*) was calculated as the ratio of the dissociation rate (*k_a_*)/association rate (*k_d_*). Considering the principle of formula $K_{D}=k_{d}/k_{a}$, the lower the *K_D_* value, the stronger the binding affinity is between analyte and mAb. Compared with the results of ic-ELISA, the differences of binding affinity between A6 and G7 were inconspicuous using a direct SPR assay against acetamiprid and thiacloprid. ACE-A6 showed a slight advantage in recognizing acetamiprid, while ACE-G7 showed a tiny margin in combining thiacloprid.

**Figure. S1**. The affinity of ACE-A6 and ACE-G7 binding thiacloprid and acetamiprid by ELISA.

**(d) ACE-G7-thiacloprid**

**(b) ACE-A6-thiacloprid**

**(a) ACE-A6-acetamiprid**

**(c) ACE-G7-acetamiprid**

**Figure. S2**. SPR kinetic sensorgrams (colored lines) and fitting curves (black lines) for ACE-A6 and ACE-G7 binding to acetamiprid and thiacloprid. The sensorgrams were fit with a 1:1 binding kinetic model.

**Table. S1**. Binding kinetic characterization of the two mAbs to acetamiprid and thiacloprid.

| Mab/pesticide | *k_a_* (1/Ms) | *k_d_* (1/s) | *K_D_* (M) | Concentrations of pesticide (nM) |
| --- | --- | --- | --- | --- |
| ACE-A6/acetamiprid | 2.145e^6^ | 2.441e^-4^ | 1.138e^-10^ | 5/2.5/1.25/0.3125 |
| ACE-A6-/thiacloprid | 3.664e^6^ | 6.547e^-4^ | 1.787e^-10^ | 5/2.5/1.25/0.3125 |
| ACE-G7/acetamiprid | 3.901e^6^ | 9.353e^-4^ | 2.398e^-10^ | 10/5/2.5/0.625/0.3125 |
| ACE-G7/thiacloprid | 1.284e^6^ | 2.627e^-4^ | 2.045e^-10^ | 5/2.5/1.25/0.625/0.3125 |

**Optimization of QDs-ICS.** According to the competitive immunoreaction principle, the concentration of ACE-OVA and QDs-Ab should be balanced. Less concentration of ACE-OVA and QDs-Ab could enhance the effect of analyte and improve the sensitivity of ICS assay. However, weak color caused by low concentrations of antigen or probe could lead to false positive results in the T line on the basis of the qualitative detection principle. The concentrations of ACE-OVA, QDs-Ab probe, and goat anti-mouse IgG should therefore be optimized by the evaluation of sensitivity and color intensity using a checkerboard test. The FI_T_ and FI_C_ values were recorded based on five repeated trials. The FI_T_/FI_C_ ratio was adjusted to offset the interference from the inherent heterogeneity of the test strips (Dzantiev et al.2014; Huang et al.2013). The competitive inhibition rates were obtained by (1-FI_T_/FI_C_)×100%. As a result, the optimum concentration of ACE-OVA was 1 mg/mL, and the optimum dilution time of QD_S_-Ab was 8000 (**Table S3**). In addition, the optimum concentration of goat anti-mouse IgG, for spraying in the NC membrane as the C line, was 0.2 mg/mL to ensure the reliability of negative samples as observed by the naked eye. Under the optimal working concentration, the means of the FI_T_ and FI_T_/FI_C_ values were 1627±147 and 1.25±0.14, respectively. The competitive inhibition rate for the 1ng/mL spiked acetamiprid standard was 69.79±5.35% (n=5) in a preset reaction time of 25min.

In the present work, the QDs-Ab probe was pre-incubated with the acetamiprid standard solution for 5 min to ensure the stability and sensitivity of ICS. Due to the high water-solubility of neonicotinoid pesticides and the side-effects of organic solvents of protein reagents, the assays were implemented in a general inorganic solvent. Subsequently, other parameters like carrier type, reaction buffer, pH value, and immunological dynamics were optimized.

The value of FI_T_ and the competitive inhibition rate were both key factors in choosing the NC membrane. As shown in **Table S4**, Sartorius CN140 (Germany) was considered the best, as it possesses an adaptive FI_T_ value of 1544±36 and an inhibition rate of 63.19±3.17% (n=5) for spraying coating antigen and a quality control antibody. The influence of the buffer was evaluated by preparing the acetamiprid standard and QDs-Ab solution for tests (n=5), and finally the intensity of fluorescence testified borate buffer (BB) was the optimal buffer (**Table S5**).

Thereafter, the effect of the pH value on the sensitivity of ICS was evaluated, by setting a series of negative solutions with pH values in the range of 5.8 to 9.0 (n=5). Obviously, the competition inhibition rate for the 1.0 ng/mL acetamiprid-spiked solution reached 68.24% of pH 7.2 (**Fig S4A**). The neutral BB (10 mM, pH 7.2) was therefore deemed the optimum reaction system for ICS.

Studying the kinetics of ICS, data were recorded every 2 minutes by the portable fluorescent reader after which it was added onto the sample pad (n=5). The kinetic curve was established by the FI_T_/FI_C_ ratio against the running time. As shown in **Figure. S4B**, the FI_T_/FI_C_ ratio could reach a stable maximum after 20 min. Thus, the optimum detection time was set as 25 min for subsequent tests.


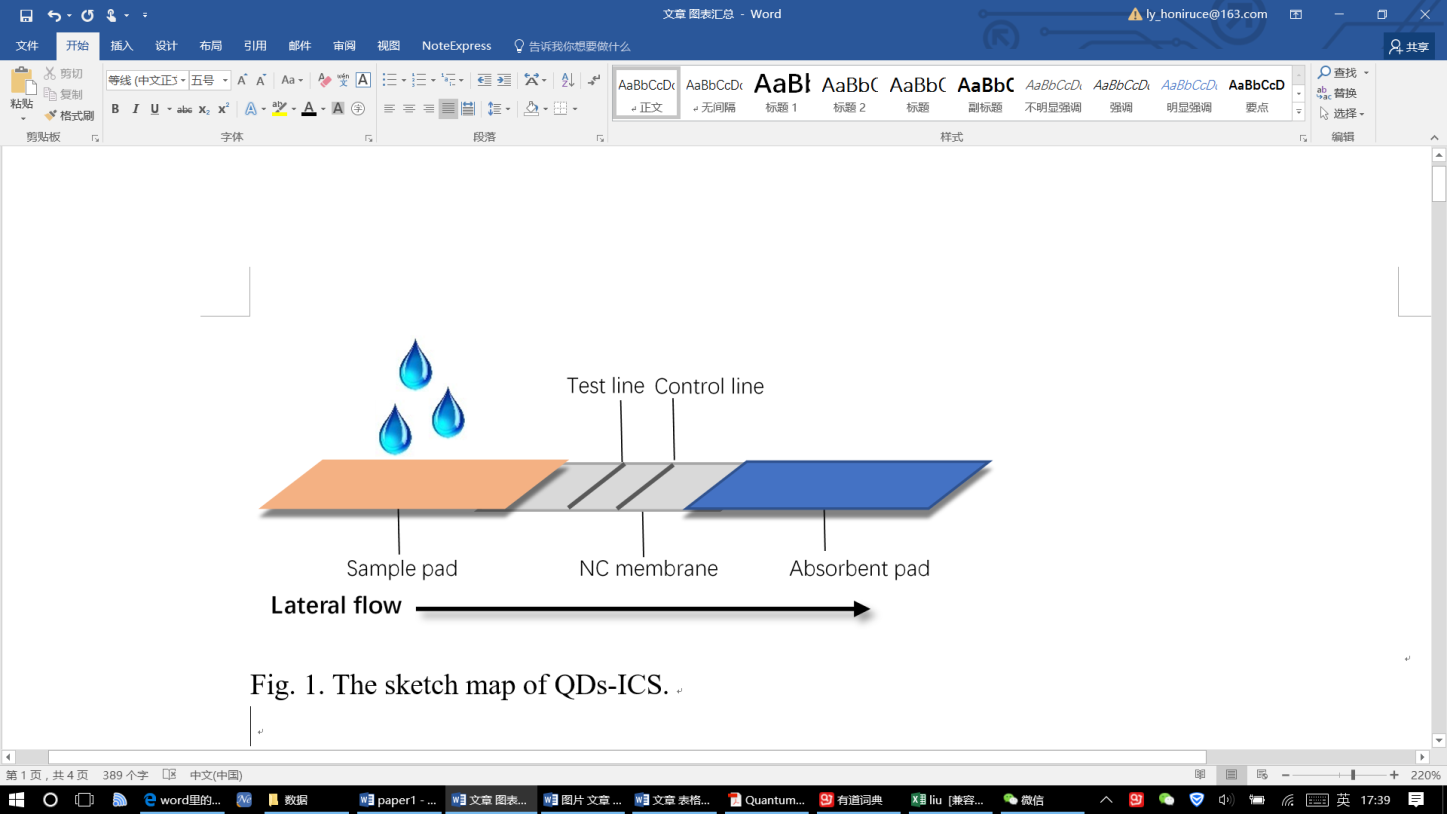

**Figure. S3**. The sketch map of QDs-ICS.

**Table. S2**. Optimization and characterization of stock solutions of QDs-Ab conjugate.

| Inclusions in stock solution | Store for three months | Effects of flowing in NC membrane | FI_T_ value in same dilutions |
| --- | --- | --- | --- |
| 0.05% NaN_3_ | Deposits | flow smoothly | 470 |
| 1% BSA + 0.05% NaN_3_ | Flocks | gathered on the border | 1063 |
| 1% BSA + 0.2% trehalose + 0.05% NaN_3_ | Uniform and transparent | gathered on the border | 1576 |
| 1% BSA + 0.05% PVP + 0.05% NaN_3_ | Flocks | flow smoothly | 866 |
| 1% BSA + 0.2% trehalose + 0.05% PVP + 0.05% NaN_3_ | Uniform and transparent | flow smoothly | 2289 |

**Table. S3**. Optimization of the concentrations of ACE-OVA and QDs-Ab by checkerboard titration.

| No. | The concentration of ACE-OVA (mg/mL) | The dilution times of QDs-Ab | FI_T_  Value | FI_T_/FI_C_ of blank control^a^ | The inhibition rate (%)^b^ |
| --- | --- | --- | --- | --- | --- |
| 1 | 2.0 | 6000 | 4628±587 | 3.56±1.02 | 43.25±16.03 |
| 2 | 2.0 | 8000 | 2721±321 | 2.06±0.15 | 52.10±9.44 |
| 3 | 2.0 | 10000 | 2122±241 | 1.69±0.18 | 56.05±9.08 |
| 4 | 1.0 | 6000 | 2160±210 | 1.73±0.35 | 64.53±7.74 |
| 5 | 1.0 | 8000 | 1627±147 | 1.25±0.14 | 69.79±5.35 |
| 6 | 1.0 | 10000 | 1185±268 | 0.98±0.18 | 56.05±9.08 |
| 7 | 0.5 | 6000 | 858±145 | 0.66±0.15 | 59.62±14.89 |
| 8 | 0.5 | 8000 | 628±107 | 0.46±0.04 | 48.91±6.79 |
| 9 | 0.5 | 10000 | 490±85 | 0.39±0.08 | 50.14±19.62 |

a. Blank control is the reaction buffer which does not contain acetamiprid standard.

b. The inhibition rates are obtained from the 1 ng/mL acetamiprid-spiked standard and the negative buffer.

**Table. S4**. The effects of NC membranes on the strip assay performance.

| Name of product | Origin | Speed (s/4 cm) | FI_T_ value^a^ | The inhibition rate (%)^b^ |
| --- | --- | --- | --- | --- |
| MDI CNPH 90 | India | 75-90 | 596±45 | 32.86±1.24 |
| MDI CNPH 150 | India | 125-150 | 706±29 | 48.51±1.06 |
| Millipore 135 | USA | 120-150 | 816±34 | 46.88±2.87 |
| Millipore 180 | USA | 160-200 | 1742±59 | 50.69±4.25 |
| Sartorius CN 95 | Germany | 90-135 | 429±13 | 36.47±3.98 |
| Sartorius CN 140 | Germany | 110-165 | 1544±36 | 63.19±3.17 |
| Pall Vivid 170 | USA | 150-225 | 1857±67 | 57.44±4.10 |

a. The fluorescence intensities of negative buffer with QDs-Ab.

b. The inhibition rates are obtained from the 1 ng/mL acetamiprid-spiked standard and the negative buffer.

**B**

**A**

**Figure. S4**. (A)The effects of pH on the inhibition radios. (B)The kinetic curve of the running time, recorded every 2 min.

**Table. S5**. The effects of buffer with various pH values.

| Buffer (10 mM) | pH | FI_T_ | FI_C_ | FI_T_/FI_C_ |
| --- | --- | --- | --- | --- |
| Borate buffer | 7.4 | 2260±112 | 2085±111 | 1.09±0.08 |
| Carbonate buffer | 9.6 | 426±63 | 1014±27 | 0.42±0.07 |
| Phosphate buffer | 7.4 | 1104±105 | 1500±61 | 0.74±0.07 |
| Tris-HCl | 7.4 | 1228±94 | 1786±93 | 0.69±0.08 |
| Deionized water | 6.4 | 956±153 | 1040±56 | 0.92±0.10 |

**Assay validation**

**Table. S6**. The matrix effect (%) in different samples dilution times.

| Sample | Dilution | 1/2 | 1/5 | 1/10 | 1/20 |
| --- | --- | --- | --- | --- | --- |
| Rice | ME (%)^a^ | 19.04 | 18.04 | 16.00 | 4.82 |
|  | FI_T_ value^b^ | 1581 | 1694 | 1697 | 1758 |
| Apple | ME (%) | 22.50 | 16.73 | 14.43 | 11.74 |
|  | FI_T_ value | 1106 | 1398 | 1401 | 1521 |
| Cabbage | ME (%) | 24.10 | 21.32 | 9.14 | -1.66 |
|  | FI_T_ value | 1308 | 1596 | 1689 | 1799 |
| Sample | Dilution | 1/50 | 1/100 | 1/200 | 1/400 |
| Green tea | ME (%) | -14.40 | -8.19 | -8.54 | 0.11 |
|  | FI_T_ value | 790 | 988 | 1278 | 1387 |
| Black tea | ME (%) | -25.55 | -19.91 | -16.67 | -7.97 |
|  | FI_T_ value | 621 | 796 | 925 | 1124 |
| Oolong tea | ME (%) | -16.90 | -12.27 | -6.47 | 2.42 |
|  | FI_T_ value | 887 | 985 | 1089 | 1277 |

a. matrix effect (%) = (1 – slope_matrix_/slope_buffer_) × 100%

b. FI_T_ value was the fluorescence intensity of T line in blank matrix extract for every dilution.

**Table. S7**. Qualifying ions and its parameters in acetamiprid detection.

| Qualifying ions (m/z) | DP (V) | EP (V) | CE (V) | CXP (V) |
| --- | --- | --- | --- | --- |
| 223→126 | 60 | 10 | 27 | 6 |
| 223→90 | 60 | 10 | 45 | 4 |


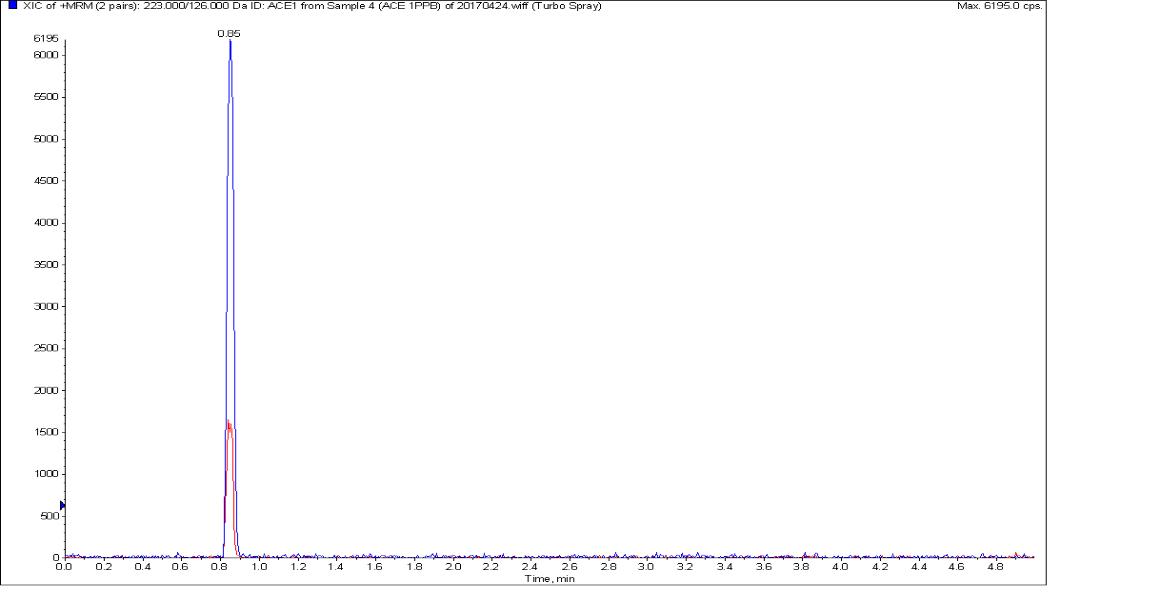


**Figure. S5**. The mass spectrogram of 1 ng/mL-spiked acetamiprid in tea matrix.

**References:**

Liu, R., Liu, Y., Lan, M., Taheri, N., Cheng, J., Guo, Y., & Zhu, G. (2016). Evaluation of a water-soluble adjuvant for the development of monoclonal antibodies against small-molecule compounds. JOURNAL OF ZHEJIANG UNIVERSITY SCIENCE B, 17(4), 282-293.

Watanabe, E. & Miyake, S. & Yogo, Y. (2013). Review of Enzyme-Linked Immunosorbent Assays (ELISAs) for Analyses of Neonicotinoid Insecticides in Agro-environments. JOURNAL OF AGRICULTURAL AND FOOD CHEMISTRY, 61(51), 12459-12472.

Dzantiev, B. B., Byzova, N. A., Urusov, A. E., & Zherdev, A. V. (2014). Immunochromatographic methods in food analysis. TrAC Trends in Analytical Chemistry, 55, 81-93.

Huang, X., Aguilar, Z. P., Li, H., Lai, W., Wei, H., Xu, H., & Xiong, Y. (2013). Fluorescent Ru(phen)32+ -Doped Silica Nanoparticles-Based ICTS Sensor for Quantitative Detection of Enrofloxacin Residues in Chicken Meat. ANALYTICAL CHEMISTRY, 85(10), 5120-5128.
